# 7-Year Follow-Up of Maxillary Overdentures Supported by Mini-Dental Implants

**DOI:** 10.3390/jcm13226891

**Published:** 2024-11-15

**Authors:** Luc Van Doorne, Laure Demeulenaere, Marie Dejans, Hugo De Bruyn

**Affiliations:** 1 Department of Plastic, Oral and Maxillo-Facial Surgery, Ghent University Hospital, 9000 Ghent, Belgium; 2 Het Tandplein, Bilkske 68, 8000 Brugge, Belgium; 3 Department of Oral Health Sciences, Faculty of Medicine and Health Sciences, Ghent University, 9000 Ghent, Belgiummarie.dejans@ugent.be (M.D.); 4 Department of Reconstructive Oral Care, Academic Centre for Dentistry Amsterdam (ACTA), Universiteit van Amsterdam and Vrije Universiteit, 1081 HV Amsterdam, The Netherlands; h.debruyn@acta.nl; 5 Faculty of Medicine and Health Sciences, School of Dental Medicine, Department of Periodontology and Oral Implantology, Ghent University, 9000 Ghent, Belgium

**Keywords:** mini-dental implant, implant survival, overdenture, oral health-related quality of life, peri-implantitis

## Abstract

**Background:** Mini dental implants (MDIs) are alternatives to support an overdenture when a standard diameter implant cannot be placed due to lack of bone volume. They reduce the need for invasive bone grafting and lower the barrier for treatment. **Aim:** This prospective study reports on implant and patient-centered outcomes of flaplessly placed, early loaded MDIs supporting horseshoe-shaped maxillary overdentures after 7 years of function. **Materials and Methods:** Patients with a CBCT-confirmed thin alveolar crest received 5–6 one-piece MDIs using mentally guided flapless surgery. The conventional denture was relined and MDIs were provisionally loaded within one week. After 6 months the non-splinted MDIs were actively loaded with a horseshoe overdenture. Implant and prosthetic survival, peri-implant health (PPD and BoP) and patient-related outcomes using OHIP-14 and Subjective Masticatory Evaluation were assessed after 7 years. **Results:** 185 MDIs were placed in 14 females and 17 males. During provisional loading, 32 MDIs were lost, and 17 replacements were required in 16 patients. In total, 170 out of 202 totally placed MDIs were supporting 29 overdentures after 6 months. Cumulative implant failure increased from 15.8% at the moment of active loading to 34.7% at 7 years. After 7 years, 76% of the initially placed overdentures remained functional, mean PPD was 3.48 (SD 0.86), BoP was 0.20 (SD 0.40) and peri-implantitis incidence was 0.9%. The total OHIP-14 was reduced from 21.3/56 (SD: 13.1) preoperatively to 15.6/56 (SD 12.8) at provisional loading (*p* > 0.1) and 7.3/56 (SD 6.7) at final loading (*p* = 0.006), and it remained unchanged up to 7 years at 6.57/56 (SD: 8.92) (*p* > 0.5). **Conclusions:** Maxillary MDIs provide an acceptable treatment option in patients with deficient bone volume that cannot or refuse to be treated with bone grafting. Despite one third of the MDIs being lost, remaining MDIs had good peri-implant health, prosthetic success was 71% and Oral Health Related Quality of Life was beyond expectations.

## 1. Introduction

Implant dentistry poses challenges when standard implants are not feasible due to limitations in bone volume or bone quality. The Cawood and Howell [1] classification describes the progressive bone resorption pattern with seven scores when the jaw becomes edentulous: score 1 being where a tooth is still present, and score 7 representing extensive alveolar bone resorption. Scores 3 and 4 represent bone which is sufficient in height but too narrow in the crestal and midfacial zone (knife edge). Scores 5 to 6 show insufficient bone height and/or width to allow conventional implant placement without additional grafting procedures.

Conventional implants are defined as implants with a diameter above 3.5 mm [2,3] and that require a bone width of at least 6 mm. Bone reconstructive procedures often require hospital surgery, are time-consuming, lead to extra cost and induce risks for morbidity such as acute or chronic pain, infection, unsteady gait, poor aesthetic results or neurologic injury [4,5]. Fear of undergoing these procedures as well as financial constraints are barriers to opting for these treatments. Reduced-diameter implants, often coined as mini dental implants (abbreviated as MDIs) may be a valuable alternative because they are indicated for thin alveolar crests [1]. In the literature, there is ambiguity about the definition of mini dental implants. In a systematic review [6], they are defined as having a diameter less than 3 mm. The ITI Consensus Conference Statement of 2018 [2] classified implants with a diameter of 3.5 mm or less as narrow-diameter implants and specifically defined “Mini implants” as having a diameter less than 2.5 mm. MDIs are used to retain overdentures [7,8,9,10]. However, the reported survival is based on short observation periods and a rather limited number of patients treated. In addition, the use of MDIs for maxillary overdenture support is rather scarce. Based on a systematic review including only 3 clinical studies [6], maxillary MDIs yield 31.7% failure with failure of 32.1% at one year [11], 22.2% at three years [9] and 33.3% at two years [12]. In a retrospective study [8,13], 5640 MDIs were examined over 12 years, with various prosthetic solutions including fixed, partial or full removable dentures. Overall survival rates for MDIs were 93.1% in the mandible and 91.3% in the maxilla, although 14.8% of MDIs failed when used to retain maxillary overdentures after three years. Several prospective reports, based on one cohort treated with six MDIs in the atrophic maxilla, yielded clinically acceptable failure rates of 17% after 2 years, 21.6% after 3 years and 23.2% after 5 years [14,15,16]. The use of MDIs may improve the retention of horseshoe designed maxillary overdentures. Patient-reported outcomes highlight enhanced satisfaction among patients undergoing this treatment [6], as well as improvement of Oral Health-Related Quality of Life [17]. Flapless implant placement has recently gained more popularity as an alternative to the classic open flap surgery and is positively evaluated by patients because it is timesaving, is less painful, is minimally invasive, and results in minor postoperative discomfort compared to the conventional open flap surgery [18,19,20]. A disadvantage of free-handed flapless surgery is that the true topography of the underlying available bone cannot be observed with higher risk of perforations and implant failure. On the other hand, when performed fully guided, higher costs are coming across [16,21]. To date, little is known of the long-term (above 5 years) clinical outcome of MDIs supporting overdentures in the maxilla.

The aim of the present clinical study is to report the implant and patient-centered outcomes of immediately loaded MDIs supporting horseshoe-shaped maxillary overdentures after 7 years of function. This includes implant and prosthetic survival, peri-implant health and patient-related outcomes, such as OHRQoL, based on OHIP-14 and subjective mastication comfort.

## 2. Materials and Methods

### 2.1. Patient Inclusion

This prospective cohort of edentulous patients in the maxilla was treated in 2 clinical centers. The subjects included were between 48 and 85 years old and fully edentulous in the maxilla. They were dissatisfied with the retention of their conventional maxillary denture. They had natural teeth in the mandible with or without a partial removable denture or had an implant-supported fixed or partial removable prosthesis. As the quantity and quality of bone determine the use of conventional implants, these MDIs were placed in patients with alveolar bone according to the Cawood and Howell classification IV [1], specifically knife-edge ridge form, adequate in height but inadequate in width. Patients refused the recommended bone augmentation procedure for reasons of fear or due to financial restrictions. Patients with metabolic diseases, systemic diseases, an immunocompromised status, previous treatment with oral or intravenous bisphosphonates and history of alveolar bone reconstruction or radiotherapy in the maxillofacial area were excluded. Smoking was not considered a contraindication for treatment. Patients received treatment over a period of 17 months, beginning in August 2015 and concluding in January 2017. Informed consent was obtained from all patients and the study was approved by the Ethic Committees of the General Hospital AZZENO Knokke–Blankenberge and the Ghent University Hospital (Belgium, registration number B670201422937).

### 2.2. Mini-Dental Implants (MDIs) 

**Figure 1:**
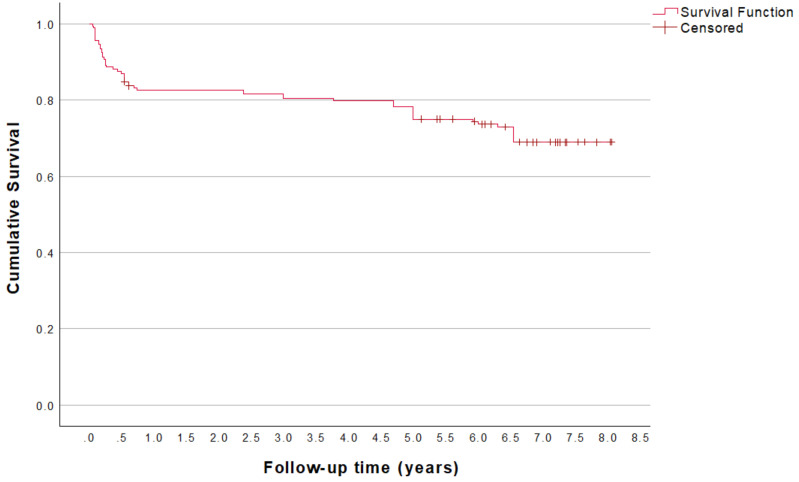
Kaplan–Meier survival analysis indicating clinical survival up to 7 years based on the originally included group of 185 MDIs (excluding replacements).

The MDI used in the study is depicted in Figure 1 (ILZ, Southern Implants Inc., Irene, South Africa) and is a one-piece, tapered implant of high-strength pure grade 4 titanium. A coronal ball measuring 1.8 mm in diameter facilitates prosthesis retention. The maximum diameter is 2.4 mm coronally, and the threaded part was 10 or 11.5 mm long. The surface roughness of the screw is moderately rough, with a Sa value of 1.5 μm. The transmucosal part, 4.8 mm long, was machined with a Sa value of 0.4 μm. 

### 2.3. Surgical and Restorative Procedure 

Prior to surgery, the existing maxillary denture with palatal coverage was relined or renewed to provide adequate fit to the soft tissues. To serve as a surgical guide, radiopaque gutta percha markers were installed in the base of the denture at the prosthetically preferred locations of the future MDIs (incisor, premolar and molar). Ideal implant placement was considered to ensure an equal load distribution with the future horseshoe overdenture. Subsequently, preoperative cone beam computed tomography was performed (Planmeca Promax 3D dental CBCT scanner, Planmeca Oy, Helsinki, Finland) with the patient wearing the denture in order to quantify the alveolar crest. Backwards planning was carried out utilizing the markers in the denture to determine the desired implant locations. To allow correct implant placement based on the CBCT information, the occlusal gutta percha fillings were removed before surgery and prepared further as drill holes.

All patients were treated under local anesthesia by one experienced maxilla-facial surgeon (LVD). Five to six MDIs were placed using a free-handed, flapless approach, using the denture for mental guidance. A triangular pilot drill (SKY-DP06/SKY-DP08, Bredent Medical, Senden, Germany) was used to penetrate both the mucosa and the alveolar cortical bone, followed by a final twisted implant drill with a diameter of 2.0 mm (ILZ D-20T; Southern Implants, Irene, South Africa). A slow-speed drilling technique with continuous sterile saline irrigation was utilized to prevent bone overheating. The drilling aimed for maximum depth, preferably reaching the cortex of the nasal or sinus floor for bicortical anchorage. Due to the flapless approach and palpation of the alveolar crest using the thumb and index finger, the drilling direction was adjusted if unintentional buccal or palatal bone perforation occurred, aiming to maintain parallelism with the other positioned implants. Implants were manually inserted, ensuring the coronal ball was positioned approximately 2 mm above the gingiva. Suturing was not required. Patients were routinely prescribed pain medication and antibiotics. Oral hygiene instructions were provided to start brushing the ball abutment parts of the MDIs twice daily with chlorhexidine gel using a soft toothbrush from day 2 postoperatively. After surgery, all patients refrained from wearing dentures for one week. At the one-week postoperative check-up, the existing denture was adjusted to fit the transmucosal ball heads using retentive soft relining material (Coesoft, GC America, Chicago, IL, USA). Patients were regularly monitored by either the surgeon or prosthodontist for relining as required. The final prosthetic connection, involving a new metal-reinforced horseshoe denture with activated attachment clips onto the ball parts of the MDI, was established six months after implant placement. Early failures during the initial healing period required replacements when denture retention was deemed compromised. 

Metal matrices with silicon caps of diverse retentive capabilities (ILZ manual, Southern Implants Inc, Irene, South Africa) were customized to fit each patient’s specific needs. The silicon caps were regularly replaced to maintain optimal retention. The lingualized occlusion concept was adopted to optimize masticatory function. 

The Appendix A gives an illustrative overview of the procedure and represents a case report with 7 years of follow-up. 

### 2.4. Clinical Examination

Patients were recalled for a 7-years clinical amination performed by 2 independent examiners. Implant and prosthetic failure (characterized by the incapacity of the existing MDIs to adequately support a horseshoe overdenture with sufficient retention) was assessed. Peri-implant health was measured. A manual pocket probe was used to measure pocket depths (PPD) mesially and distally with minimal pressure. The average of these 2 values was calculated per implant. Consecutively, bleeding on probing (BoP) was measured mesially and distally. In the case of bleeding on one or two sites, the respective implant received a dichotomous score of 1. Peri-apical radiographs were not considered reliable. Given the extreme resorption and non-parallel direction of the MDIs in the crest, the correct direction of the röntgen beam was hampered. Therefore, peri-implantitis on the implant level was diagnosed based on a pocket depth greater than or equal to 6 mm in combination with one or two bleeding sites.

### 2.5. Patient-Reported Outcome Measures

The validated Dutch version of the OHIP-14 questionnaire was used to assess the Oral Health-Related Quality of Life (OHRQoL) [22]. This questionnaire assesses seven domains, with two questions allocated to each domain, making a total of 14 questions. The domains are defined as functional limitation, physical disability, physical pain, psychological disability, psychological discomfort, social disability and handicap. The five response categories for each item are as follows: never (=0), hardly ever (=1), occasionally (=2), fairly often (=3) and very often (=4). The domain score is calculated as the mean of the two specific domain questions, hence ranging from 0 to 4. The Total OHIP-14 is calculated as the sum of responses for each item. A maximum positive score of 0 indicates a high quality of life, while a maximum negative score of 56 indicates a low quality of life. The results after 7 years were compared with previous registrations taken preoperatively, postoperatively, after active loading and during yearly follow-up visits. In addition, the Subjective Masticatory Evaluation was evaluated based on 5 questions. Patients were asked to estimate their masticatory performance to chew a piece of soft white bread, hard cheese, dry sausage, apple and carrot on a 100 mm scale [23]. Those 5 Visual Analogue Scale (VAS) responses were summed with a maximum score of 50. The lower the value, the better the chewing perception. 

### 2.6. Statistical Analysis

Statistical analysis was performed using SPSS version 28 (IBM SPSS, statistics for Windows, version 25.0, Business Analytics, Amonk, NY, USA). The Kaplan–Meier survival analysis was used for the overall MDI survival as well as related to gender and smoking habit. The dependent Samples T-test was used to compare OHIP-14 changes over time.

## 3. Results

### 3.1. Patients

The initial study included 31 subjects: 14 females (45.2%) and 17 males (54.8%) with a mean age of 62.30 years (range: 48–83; SD 9.28); 9 patients (29.0%) persisted in smoking. At the 7-year recall, 7 patients were lost to follow-up and considered dropouts, and 24 patients remained, of which 3 were lost to follow-up. Eleven females (54.2%) and thirteen males (45.8%) with a mean age of 70.04 years (SD 9.072) were included in the final analysis. Of these, 18 patients (75%) had a natural antagonist dentition, 3 (12.5%) had a combination of a partial denture and natural teeth and the remaining 3 (12.5%) had an implant-supported overdenture. 

### 3.2. Clinical Outcomes

Overall, 185 MDIs were initially placed in 31 patients. During the provisional loading period, 32 MDIs (17.3%) were lost in 16 patients, and 17 MDIs were replaced. Two patients with five failures each opted for another implant solution and were dropouts. Including the retreatments, 204 MDIs were placed in total, and 170 MDIs were actively loaded with 29 overdentures. During the 5-year follow-up period, 14 additional MDIs were lost in three patients, all with a history of previous MDI failure, and 2 MDIs were replaced in one patient. This yielded a 5 years’ cumulative survival implant failure rate of 23.2%. At the 5year interval, one patient was deceased, and two patients were lost to follow-up. Hence, 26 patients remained for longer follow-up. Between 5 and 7 years, 8 MDIs were lost and three patients with 18 MDIs were dropouts. After 7 years, one patient had six MDIs removed due to poor functionality, opting for an alternative implant treatment. Hence, 22 overdentures (75.9%) of the initially placed 29 remained functional. Details are presented in Table 1. Kaplan–Meier analysis of the originally placed 185 MDIs is shown in Figure 1. 

**Figure 2:**
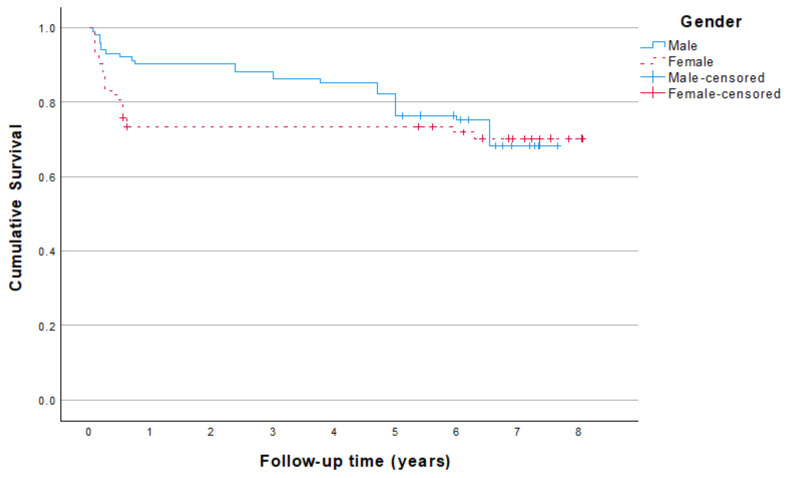
Kaplan–Meier survival analysis in relation to gender up to 7 years. Based on 185 originally placed mini dental implants; replacements have been excluded.

**Figure 3:**
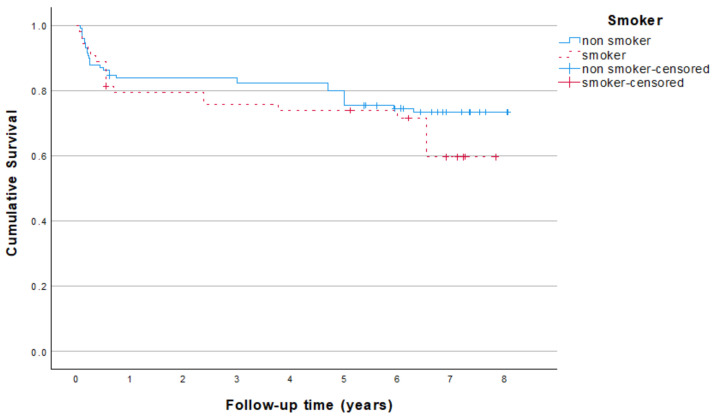
Kaplan–Meier survival analysis in relation to smoking habit. Based on 185 originally placed mini dental implants; replacements have been excluded.

Subgroup analysis related to gender and smoking is depicted in Figure 2 and Figure 3.

### 3.3. Peri-Implant Health

This outcome involved data from 20 patients and 109 MDIs. Two missing patients could not attend due to medical reasons but were contacted by phone to receive information on the implant status. Hence, these cases were included in the survival analysis. The mean PPD and BoP for 109 implants at 7 years was 3.48 (SD 0.86; range 2–6.5) and 0.20 (SD 0.40; range 0–1), respectively; 20.2% of the MDIs showed BoP. Only one MDI (1/109) showed peri-implantitis (0.9%). The detailed results are shown in Table 2.

### 3.4. Patient-Reported Outcomes

**Figure 4:**
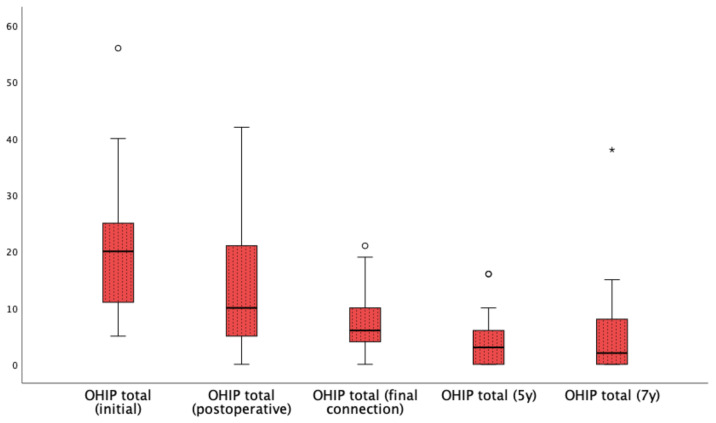
Boxplot of total OHIP-14 scores at the follow-up intervals. ° outlier, * extreme.

Patient-reported outcomes were assessed by means of the OHIP-14 and Subjective Masticatory Evaluation VAS in 23 subjects. The mean total OHIP-14 score after 7 years was 6.57/56 (SD 8.92; range 0–38). Table 3 and Figure 4 show the changes in total OHIP-14 score at the different follow-up intervals. The mean domain scores at different moments of data collection are presented in Table 3. A slight, not statistically significant, decrease in the mean total OHIP-14 score is observed from baseline (with the original denture prior to implant placement) to provisional loading (postoperative). Furthermore, a statistically significant (*p* = 0.006) decrease was observed after final active prosthetic connection, and this remained unchanged up to 7 years.

The subjective masticatory evaluation was assessed in 23 subjects. This included the patient in which all six MDIs were removed afterwards due to poor functionality. The mean total score of the Subjective Masticatory Evaluation after the 7-year follow-up was 15.18 (SD 12.46). The minimum total score was 1.6, and the maximum was 49.1. Overall, the capability of chewing a piece of carrot scored the worst with a mean score of 3.81 (SD = 3.11). The different subcategories are presented in Table 4.

## 4. Discussion

This prospective 7-year follow-up study evaluated MDIs placed with flapless surgery and early non-functional loading for supporting a maxillary horseshoe overdenture. The findings reveal various aspects of implant survival, prosthetic success, peri-implant health and patient-reported outcomes. The treatment protocol is challenging because it offers a minimally invasive and cost-effective solution for edentulous patients with insufficient bone volume or quality in the maxilla in addition to financial constraints or barriers for extensive maxillofacial surgery. 

The study revealed multiple MDI failures during the initial healing phase probably related to the early loading with a relined denture. This necessitated multiple replacements to ensure stability of the overdentures in cases of multiple MDI failures. After 2 years, this yielded 17% MDI failures, which is line with two other retrospective studies reporting overall survival rates for MDIs of 91.3% in the maxilla but 14.8% failures when MDIs were used to retain a maxillary overdenture after three years [9,13]. After 7 years, the cumulative survival of the present study was 70% for the MDIs and 76% for the prosthesis. This outcome is comparable with the failure of 31.7% in the report by Lemos et al. [6]. The lower survival rate of maxillary MDIs compared to conventional dental implants can be attributed to a combination of factors, including compromised maxillary bone conditions, the free-handed flapless surgical protocol and early loading, albeit non-functionally, after one week. In this respect, one should note that women initially presented with higher early failure rates [14]. This could be related to the likelihood of osteoporosis or the fact that females did not follow our advice to refrain from denture-wearing overnight. 

The survival rate of MDIs could potentially be improved by refining preoperative diagnostics and using fully guided surgery instead of free-handed flapless. Despite the use of the denture as guide plate, this protocol can be considered as mentally guided because the surgeon was not strictly guided in bone as is the case when a drill guide is used. In the current study, this was adopted for financial reasons. Future research should investigate whether the outcome can be improved by being strict in choosing the implant direction. Indeed, a 2-year CBCT analysis of the same cohort revealed multiple perforations of the alveolar crest as well as nose or sinus. The authors suggested this may have influenced initial implant stability and early osseointegration [15]. 

The prevalence of peri-implantitis, arbitrarily chosen as a combination of BoP and PPD equal to or above 6 mm, was low (0.9%). This is a positive outcome, as peri-implantitis can lead to implant loss and negatively impact patient satisfaction and Oral Health-Related Quality of Life [16]. A possible reason can be found in the design of mini dental implants. There is no microgap because it is designed as one-piece implant with dimensions not allowing a screw-retained abutment component. It is known that a microgap is a potential site for bacterial accumulation and inflammation due to microleakage. Furthermore, micromovements between an abutment and a conventional fixture are proven to have a negative effect on the crestal bone. The prevalence of BoP is 20% in the current study. This is extremely low in comparison with two-piece implants [24,25,26,27]. The BoP status may be affected by the inclusion of smokers. Smoking has a masking effect on inflammation, making it challenging to assess peri-implant health accurately in smokers. The degree of bleeding may not correlate with the severity of inflammation in smokers. It is essential to consider this factor when interpreting peri-implant health outcomes, as smoking has been identified as a potential contributing factor to peri-implantitis [28].

Previous studies have shown that MDI-supported overdentures in the maxilla can improve retention and patient satisfaction [6,14,17], making them a valuable alternative to conventional implants. Patient-reported outcomes, assessed through OHIP-14 and Subjective Masticatory Evaluation VAS, showed a gradual improvement in Oral Health-Related Quality of Life and masticatory function over the follow-up period, and this occurred despite multiple MDI failures. In a previous report, Van Doorne et al. [17] demonstrated that a failure of one MDI did not affect the OHIP-14 score, but in the case of multiple failures, OHRQoL did not improve. For this reason, faster replacements of lost MDIs are recommended. It is likely that this could also have prevented future losses. Indeed, supporting the overdenture on six MDIs ideally distributed on the alveolar crest would have enhanced optimal loading and nice loading force distribution. Replacing a lost MDI is a simple, cheap and non-invasive procedure which is more cost beneficial than losing more implants, eventually leading to loss of overdenture function with a dissatisfied patient as result. Premature loading of the non-connected MDIs could also affect the failure rate. Connecting the mini-implants with a bar could possibly reduce the risk of early failures.

Our study has a few limitations that need to be recognized. Firstly, the absence of a control group, like in an RCT study design, restricts our ability to compare outcomes against a standard or alternative treatment. It is, however, obvious that MDI failures are significantly higher than with conventional implants predominantly because of a negative case selection. In our opinion, an RCT is not an option because MDIs are placed in crestal bone with insufficient width for normal conventional implants. However, it would be worthwhile to compare the current treatment protocol with protocols advocating extensive bone graft surgery. Secondly, the limited sample size, related to dropouts, resulted in a restricted number of cases being followed over time, which may potentially impact the generalizability of our findings. 

## 5. Conclusions

After a 7-year follow-up, atrophic edentulous maxilla rehabilitation with a six MDI-supported overdenture, in the case of non-functional conventional denture, improves patients OHRQoL, despite reduced implant survival. It is crucial to inform patients about higher potential implant losses, especially in the long term, for managing expectations and ensuring patient satisfaction. Future research is required to refine treatment protocols by optimizing flapless surgery techniques, increasing the number of MDIs, achieving early inter-implant fixation of the MDIs and compensating and preventing potential MDI losses over time. Last but not least, a mind change in clinicians is required when selecting edentulous patients with financial and anatomical constraints. It is not the bone that sets the tone but the patient-reported quality of life.

## Figures and Tables

**Table 1:** Clinical outcome related to installed, reinstalled and lost MDIs for 7-year follow-up.

	MDIs Start Interval (N)	MDIs Lost (N)	MDIs Unaccounted for (N)	MDIs Reinstalled (N)	MDIs End Interval	Cumulative Failure (%)	Prostheses at End Interval
Baseline to functional loading	185 (31)	32 (16)		17 (10)	170	15.8	29
Functional loading up to 2 years	170 (29)	4 (1)			166	17.8	29
25–36 months	166 (29)	4 (2)		2 (1)	164	19.8	29
37–48 months	164 (29)	3 (1) ^a^			161	21.7	28
49–60 months	161 (28)	3 (1) ^a^	6 (1) ^b^		152	23.2	26
61–72 months	152 (27)	1 (1)	18 (3) ^c^		133	26.1	23
73–84 months	133 (24)	7 (2) ^a,d^			126	30.0	22

^a^ Implant failure led to prosthetic failure; ^b^ Patient deceased; ^c^ 3 patients lost to follow-up; ^d^ 6 MDIs of the remaining 7 were removed due to poor functionality, opting for an alternative implant treatment.

**Table 2:** BoP in function of PPD (mm) after 7 years.

	PPD after 7 Years (mm)										
	2.0	2.5	3.0	3.5	4.0	4.5	5.0	5.5	6.0	6.5	Total
no BoP	5	10	25	19	12	13	0	1	1	1	**87**
BoP	0	3	8	3	3	3	1	0	1	0	**22**
**Total**	**5**	**13**	**33**	**22**	**15**	**16**	**1**	**1**	**2**	**1**	**109**

**Table 3:** (**A**) Total OHIP-14 score at different moments of data collection; (SD); ((N)): number of cases. (**B**) Mean OHIP Domain scores, (SD), Scale from 0–4, Domain names: functional limitation (F_LT), physical disability (Phys_DA), physical pain (Phys_P), psychological disability (Psych_DA), psychological discomfort (Psych_DC), social disability (S_DA) and handicap (H).

**A.**	**Total OHIP-14**	**Initial**	**Postop**	**Final Connection**	**3 Years Function**	**5 Years Function**	**7 Years Function**
	Score (SD) ((N))	21.3 (13.1); ((31))	15.6 (12.8); ((29))	7.3 (6.7); ((29))	6.5 (8.9); ((28))	4.96 (5.00); ((27))	6.57 (8.92); ((23))
	*p*-value compared to previous interval		*p* > 0.1	*p* = 0.006	*p* > 0.5	*p* > 0.1	*p* = 0.09
**B.**	**DOMAINS**	**Initial**	**Postop**	**Final connection**	**3 years** **function**	**5 years** **function**	**7 years** **function**
	F_LT	1.56	0.74 (0.69)	0.62 (0.75)	0.52 (0.60)	0.39 (0.51)	0.45 (0.54)
	Phys_DA	1.77 (1.10)	0.67 (0.67)	0.66 (0.72)	0.56 (0.51)	0.65 (0.62)	0.74 (0.94)
	Phys _P	2.10 (1.23)	0.47 (0.64)	0.67 (0.96)	0.56 (0.82)	0.43 (0.69)	0.59 (0.91)
	Psych_DA	1.29 (1.21)	0.45 (0.72)	0.22 (0.45)	0.26 (0.45)	0.26 (0.51)	0.52 (0.95)
	Psych _DC	1.65 (1.13)	0.52 (0.62)	0.47 (0.75)	0.39 (0.59)	0.26 (0.42)	0.46 (0.69)
	S_DA	0.97 (1.05)	0.34 (0.55)	0.36 (0.83)	0.17 (0.39)	0.18 (0.33)	0.24 (0.40)
	H	0.56 (0.94)	0.34 (0.57)	0.19 (0.60)	0.15 (0.33)	0.35 (0.48)	0.29 (0.60)

**Table 4:** Results of the different subjective masticatory subcategories, on a VAS scale from 0 to 10.

	Mean	Std. Deviation	Minimum (cm)	Maximum (cm)
Capability to chew				
Soft white bread	2.091	2.437	0.10	10.0
Hard cheese	2.733	2.615	0.20	9.90
Dry sausage	2.872	2.388	0.30	9.70
Apple	3.674	3.178	0.30	10.0
Carrot	3.813	3.107	0.40	10.0

**Figure A1:**
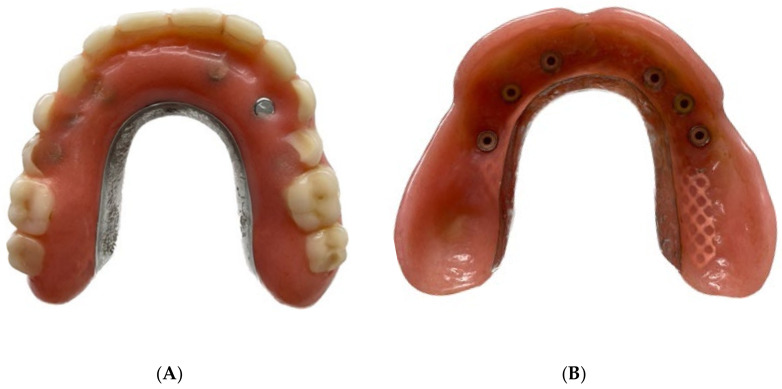
(**A**,**B**) Metal-reinforced horseshoe denture featuring metal matrices with silicon clips of different retentive capabilities (ILZ manual, Southern Implants Inc, Irene, South Africa) to acquire attachment onto the ball parts of the MDI.

**Figure A2:**
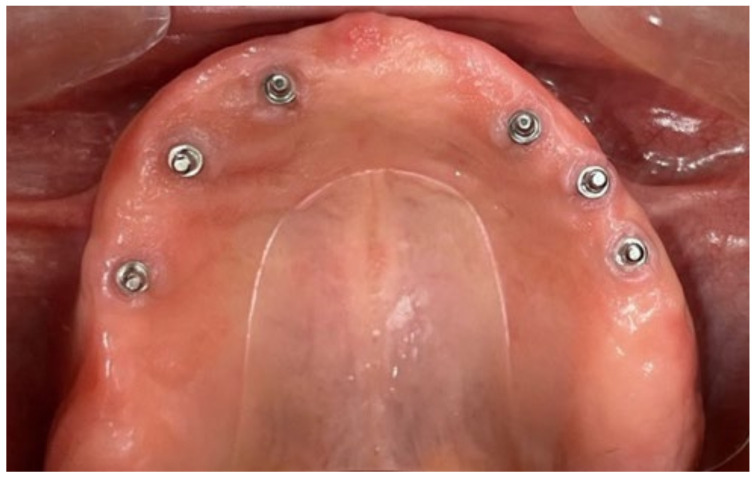
The 6 MDIs in situ with the coronal ball attachment. Pinpoint abrasion and deformation of the coronal ball can be observed after 7 years.

**Figure A3:**
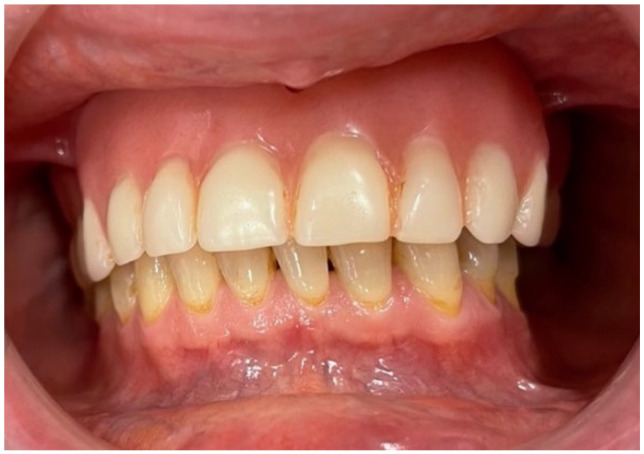
The denture in occlusion after attachment onto the MDIs. This case had a natural dentition as antagonist.

**Figure A4:**
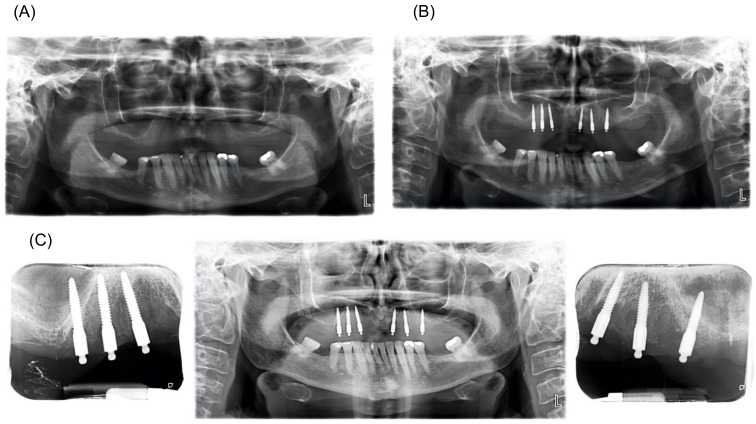
(**A**) The radiograph after extraction, preoperative. (**B**) The radiograph after implantation, postoperative. (**C**) The radiographs after 5 years of function (panoramic X-ray and detailed periapical images).

## Data Availability

Data is contained within the article.
